# Thermal Safety of Forced-Air Warming During Balloon Occlusion in Isolated Perfusion Chemotherapy: A Prospective Feasibility Study Using Multisite Temperature Monitoring

**DOI:** 10.3390/cancers18101640

**Published:** 2026-05-19

**Authors:** Hansjoerg Aust, Peter Kranke, Alexander Torossian, Kornelia Aigner

**Affiliations:** 1 Medias Klinikum Burghausen, Department of Anaesthesiology, 84489 Burghausen, Germany; 2 University Hospital Giessen and Marburg (UKGM), Clinic of Anaesthesiology and Intensive Care Medicine, 35043 Marburg, Germany; torossia@med.uni-marburg.de; 3 University Hospital Würzburg, Department of Anaesthesiology, Intensive Care, Emergency and Pain Medicine, 97080 Würzburg, Germany; kranke_p@ukw.de; 4 Medias Klinikum Burghausen, Department of Tumour Biology, 84489 Burghausen, Germany; kornelia.aigner@medias-klinikum.de

**Keywords:** isolated perfusion chemotherapy, forced-air warming, perioperative hypothermia, temperature monitoring, balloon occlusion, ischemia–reperfusion

## Abstract

Background: Isolated Perfusion Chemotherapy (IPC) with balloon occlusion creates transient ischemic tissue compartments while patients remain exposed to significant perioperative heat loss. Active warming during these phases is commonly avoided due to theoretical concerns regarding impaired heat distribution and potential local heat accumulation in ischemic tissue. This study investigated the thermal safety of forced-air warming during IPC under these conditions. Methods: In this prospective observational study, 31 patients undergoing IPC were monitored during balloon-induced vascular occlusion. Convective warming was applied using a forced-air system set to 43 °C. Core temperature was measured rectally, and local temperatures were continuously recorded at gluteal, lumbar, and interscapular sites. Temperature trajectories and maximum values during occlusion were analysed descriptively. Results: Local temperature increases during ischemia were limited, with a maximum increase of 2.3 °C at the lumbar site. Absolute temperatures remained well below the predefined safety threshold of 39.5 °C at all skin measurement sites (maximum observed 37.7 °C). Core temperature remained stable throughout the occlusion phase. No evidence of local heat accumulation, threshold exceedance, or thermal skin reactions was observed. Conclusions: Under conditions of controlled application, close temperature monitoring, and short ischemic intervals, forced-air warming during IPC did not result in local overheating or clinically relevant thermal exposure. These findings challenge the prevailing precautionary approach of avoiding active warming during vascular isolation and provide prospective clinical evidence supporting a reassessment of temperature management strategies toward actively maintained normothermia in isolated perfusion chemotherapy.

## Simple Summary

During isolated perfusion chemotherapy, blood flow to certain parts of the body is temporarily stopped to deliver high-dose chemotherapy more precisely while reducing side effects. However, patients are at risk of losing body heat during this procedure. Active warming is commonly used in surgery but is often avoided in this setting due to concerns that heat could build up in areas with reduced blood flow and may cause tissue damage. In this study, we investigated whether active warming is safe under these conditions. We monitored body temperature in 31 patients at multiple sites, including core and skin temperature measurements. Our results showed no harmful temperature increases or signs of tissue injury. These findings suggest that active warming can be safely used to prevent hypothermia, even when blood flow is temporarily interrupted. This may improve temperature management and patient safety during this type of cancer treatment.

## 1. Introduction

Isolated Perfusion Chemotherapy creates a fundamental thermophysiological dilemma with direct implications for oncological treatment safety. While active warming is required to prevent perioperative hypothermia, balloon occlusion simultaneously induces transient ischemia in which physiological heat distribution is impaired. This raises unresolved safety concerns regarding potential local heat accumulation and tissue injury during cancer therapy.

**Figure 1:**
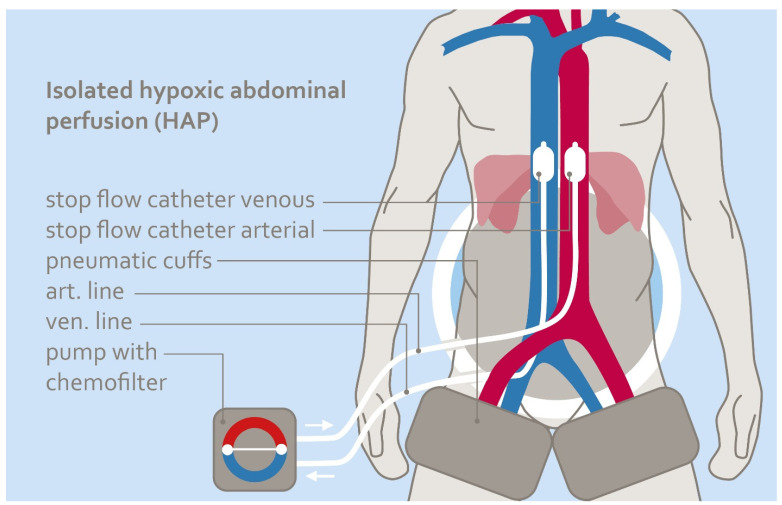
Schematic illustration of Isolated Perfusion Chemotherapy (IPC) with balloon occlusion and extracorporeal circulation. Red and blue indicate arterial and venous circulation, respectively, while the white outlined area represents the isolated perfusion region.

The technique of Isolated Perfusion Chemotherapy (IPC) (see Figure 1) represents an extension of isolated chemotherapeutic organ perfusion techniques. It started with isolated viscera and limb perfusion, which was first introduced by Edward Krementz and George Creech in 1957 at Tulane University [1,2]. Over the following years, the approach was expanded to include isolated perfusion of internal organs such as the liver, with the basic concept remaining unchanged: to deliver highly concentrated cytotoxic agents to a single limb or organ while limiting systemic exposure [3,4,5]. Since then, the technique has been further developed and extended to larger anatomical regions in order to better target locally advanced malignancies, including primary tumors, lymphatic spread, and regional metastases (see Table 1) [6,7,8,9].

In IPC, vascular isolation of the targeted anatomical compartment is achieved by temporary balloon occlusion of the aorta and vena cava. Regional circulation within the isolated territory is maintained via extracorporeal circulation, while hemofiltration and protective measures such as head cooling are applied simultaneously to limit systemic exposure to the chemotherapeutic agents [10,11,12].

During balloon occlusion, the affected body compartment undergoes transient ischemia. At the same time, patients are exposed to substantial heat loss caused by extracorporeal circulation, hemofiltration, and head cooling protocols used to reduce systemic toxicity. As a result, clinically relevant perioperative hypothermia may occur and often requires active management to maintain physiological stability. Perioperative hypothermia is associated with impaired coagulation and increased blood loss, a higher risk of cardiac complications, and prolonged recovery due to altered drug metabolism. In addition, shivering should not be regarded merely as discomfort, as it can markedly increase metabolic rate and oxygen consumption, thereby adding relevant systemic stress in patients already exposed to cytotoxic treatment and extracorporeal circulation and transient ischemia [13,14,15,16,17].

In procedures involving isolated regional perfusion, temperature regulation can be particularly challenging due to altered perfusion dynamics and redistribution of body heat. In clinical settings without structured warming strategies, transient decreases in core body temperature have been observed during these procedures, highlighting the importance of targeted temperature management. The implementation of standardized warming strategies has been associated with more stable temperature profiles.

Active warming is widely recommended to prevent perioperative hypothermia according to current guidelines [18,19]. However, its use during periods of impaired perfusion or ischemia raises theoretical safety concerns. In perfused tissue, heat distribution is largely mediated by blood flow. During ischemia, impaired perfusion may theoretically lead to local heat accumulation and tissue injury. Similar considerations have been discussed in the context of tourniquet physiology and warm ischemia [20,21].

Evidence addressing the safety of active warming during transient ischemia remains scarce, both in IPC and in comparable clinical settings involving temporary vascular occlusion or impaired perfusion. Consequently, the thermal safety of convective warming under these conditions remains insufficiently characterized.

The present study was therefore designed as a pragmatic feasibility and safety assessment to address this unresolved clinical dilemma. Rather than aiming to provide comparative or inferential evidence, the study specifically evaluates whether continuous convective warming during balloon-induced vascular occlusion leads to (1) clinically relevant local heat accumulation at skin–device contact zones and (2) unintended increases in core temperature. By focusing on these safety endpoints under real-world clinical conditions, the study aims to provide an initial evidence base to inform future investigations.

## 2. Methods

### 2.1. Study Design and Rationale

This prospective observational feasibility study investigated the thermal safety of uniform convective warming during IPC with balloon-induced vascular occlusion. The study was initiated following repeated observations of significant perioperative heat loss during these procedures and was designed to systematically evaluate potential thermal risks associated with warming during transient ischemia.

Following a review of the literature and interdisciplinary consultation with experts from departments of anesthesiology at university hospitals in Marburg and Würzburg, Germany, a structured safety-oriented observational study was designed. The protocol focused on feasibility under routine clinical conditions while incorporating predefined safety monitoring and detailed local temperature measurements to detect potential thermal extremes.

Particular emphasis was placed on continuous central and local temperature monitoring and on predefined safety thresholds that allowed early identification of potentially harmful temperature elevations and immediate reassessment of the warming strategy if required.

To complement intraoperative monitoring, all patients underwent a standardized postoperative clinical examination on the evening of surgery before night rest. The examination specifically assessed early signs of thermal skin reactions at pressure or contact zones exposed to warming, including erythema, irritation, or patient-reported discomfort.

### 2.2. Patient Population and Sample Size

Consecutive adult patients undergoing IPC in thoracic, abdominal, or pelvic settings were included. The sample size was defined based on safety considerations. An initial cohort of 30 patients was planned to capture potential thermal extremes. Following internal safety review of the accumulated data, recruitment continued consecutively, resulting in a final study population of 31 patients.

Given the theoretically highest ischemic risk, analyses were predefined to focus on thoracic and abdominal procedures, while pelvic procedures were recorded and analyzed descriptively.

### 2.3. Procedure and Ischemic Phases

Isolated perfusion was performed using balloon occlusion according to an established clinical protocol. Depending on the anatomical setting, different ischemic phases occurred:

- Thoracic perfusion: complete occlusion with cessation of perfusion to body regions below the diaphragm for approximately 15 min (longest period) during the chemotherapy phase.
- Abdominal perfusion: complete vascular occlusion for approximately 5 min, followed by regional isolated perfusion via connection to an extracorporeal circuit. Balloons were deflated after a total occlusion time of 15 min.
- Pelvic perfusion: identical protocol, with complete vascular occlusion for approximately 5 min followed by regional isolated perfusion via extracorporeal circulation. Total occlusion time was 15 min.

Temperature monitoring was conducted in a standardized manner across all procedures.

### 2.4. Antineoblastic Management

All IPC procedures were performed using antineoblastic drugs. In the majority of cases (>90%), a cisplatin-based polychemotherapy regimen was applied. The specific drug combinations and doses were adapted to prior chemotherapy exposure, organ function, and overall clinical status. In patients with relevant pre-existing organ impairment, particularly renal dysfunction, dosing and/or exposure time were adjusted accordingly. These variations did not affect the perfusion technique or the anesthesiological management, including temperature control.

### 2.5. Anesthetic Management

All procedures were conducted under total intravenous anesthesia (TIVA) using propofol and remifentanil. Anesthesia and hemodynamic management followed a strict institutional standard operating procedure (SOP). Norepinephrine was administered as needed and titrated individually to maintain a mean arterial pressure of at least 60 mmHg. Transient systolic blood pressure increases during balloon occlusion were tolerated up to 160 mmHg; higher values were treated with intravenous nitroglycerin boluses. Standard antiemetic prophylaxis was given with ondansetron. Operating room temperature was kept at approximately 20 °C. Patients with a core body temperature below 34 °C were rewarmed to at least 35.5 °C before termination of anesthesia, resulting in a corresponding prolongation of anesthesia. Airway management was routinely performed using a second-generation supraglottic airway device (laryngeal mask airway, LMA, London, UK); endotracheal intubation was reserved for patients with specific indications, such as known gastroesophageal reflux.

### 2.6. Anticoagulation and Coagulation Monitoring

Systemic anticoagulation was standardized using unfractionated heparin (initial bolus: 150 IU/kg body weight) followed by ACT-guided management during extracorporeal circulation and protamine reversal as clinically indicated.

### 2.7. Extracorporeal Circulation and Hemofiltration

Extracorporeal circulation was used during the isolated perfusion phase and subsequently for hemofiltration to remove chemotherapeutic agents from the systemic circulation. Head cooling and hemofiltration were applied according to the routine clinical protocol of the center.

### 2.8. Warming Management

Convective warming was applied in all patients using a forced-air warming system (Möck Warming System^®^, type EW II, Möck & Möck Medizintechnik, Hamburg, Germany) in combination with a Twinwarm BB forced-air device (type 12BB-DE-EF, Möck & Möck Medizintechnik, Germany).

A large-area warming mat with upper-body overlay was positioned uniformly from the inguinal region to the shoulder area in order to maximize heat transfer to the central body compartments. Previous studies have shown that heat donation applied to the torso provides effective systemic heat transfer during rewarming compared with more peripheral warming strategies [22]. The system’s outlet temperature was standardized at 43 °C and maintained throughout the entire procedure, including the occlusion phase.

### 2.9. Temperature Measurement

Temperature monitoring was performed centrally and locally using standardized sensor technology.

Core body temperature was continuously measured using a rectal temperature probe (General Purpose Temperature Probe Adult, B. Braun, Melsungen, Germany) and served as the reference measure for central body temperature. Esophageal and nasopharyngeal monitoring, although generally preferred for perioperative core temperature assessment [15,23], were not suitable in the present setting because esophageal probes interfered with radiographic imaging and nasopharyngeal measurements were potentially confounded by concomitant head cooling. Intravascular monitoring was not indicated. Rectal measurement was therefore selected as the most feasible reference method under these conditions. This approach was also chosen to reflect a monitoring strategy that can be consistently implemented in routine clinical practice beyond the study setting.

Local skin and contact temperatures were measured using contact sensors (T-Skin^®^, B. Braun, Germany) placed at three predefined support zones with direct contact to the warming system and therefore the highest theoretical risk for local heat accumulation:

- gluteal region at the deepest support point
- lumbar region paravertebrally at the level of the lumbar spine
- interscapular region between the shoulder blades

The contact sensors were deliberately not thermally isolated from the underlying warming surface. This methodological choice was made to capture the effective temperature at the patient–support interface under real clinical conditions, rather than an artificially isolated skin temperature. The aim was to assess potential local heat accumulation in areas of direct contact with the warming device, where thermal injury would be most likely to occur in the presence of reduced perfusion. Accordingly, the recorded values represent interface-relevant temperatures reflecting the combined effects of skin temperature, contact pressure, and local heat transfer dynamics.

In addition, upper-body surface temperature was assessed using an infrared thermometer (FTW-01, Phicon Biotech Co., Ltd., Guangzhou, China) as a supplementary non-contact measurement to characterize the superficial thermal environment at the upper body surface, particularly with regard to heat accumulation beneath the surgical drapes.

**Figure 2:**
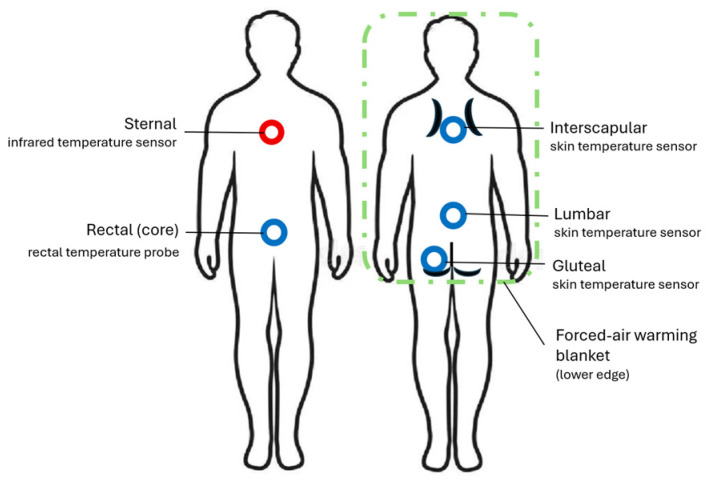
Measurement sites and warming area used for temperature monitoring.

In total, five temperature measurement sites were recorded simultaneously (see Figure 2). Measurements were obtained continuously or at short intervals during the occlusion phase, documenting temperature trajectories and maximum recorded values.

### 2.10. Safety Definition and Alarm Threshold

To ensure patient safety, predefined conservative thermal thresholds were specified a priori in the study protocol, differentiating between peripheral (skin) and core temperature.

For skin temperature, a threshold of ≥39.5 °C at the measurement sites (gluteal region, lumbar region, and interscapular region) was defined as a pragmatic and conservative safety limit to prevent local heat accumulation and potential thermal injury at the interface between the heating device and the skin [24]. This threshold was intentionally selected well below temperature ranges associated with thermal tissue damage reported in the literature [16,25,26], and therefore represents a precautionary margin rather than a physiological injury threshold. In the absence of established reference values for this specific clinical setting, the threshold was defined a priori as an operational safety trigger to allow early detection of potentially unintended temperature increases and to prompt immediate reassessment of the warming strategy.

For core temperature, assessed rectally, a maximum threshold of 37.5 °C was defined. This limit was selected to avoid unintended systemic or regional heat exposure in the context of reduced perfusion and hypoxic conditions, while still allowing controlled prevention of clinically relevant hypothermia [27].

Both thresholds were defined pragmatically based on physiological considerations and clinical safety margins in the absence of established reference values for this specific setting.

All study personnel were instructed regarding the predefined alarm thresholds and corresponding response procedures prior to study initiation. If either threshold was reached or exceeded, immediate reassessment of the warming strategy was planned.

### 2.11. Endpoints and Data Analysis

The study defined two co-primary safety endpoints: (1) the maximum local skin temperature measured at the predefined sites during the occlusion phase, serving as a surrogate marker for potential local heat accumulation at the skin–device interface, and (2) the maximum rectal core body temperature, reflecting systemic thermal exposure under conditions of reduced perfusion. Both endpoints were assessed against their respective predefined safety thresholds (≥39.5 °C for skin temperature and ≥37.5 °C for core temperature). Exceedance of either threshold was defined as a safety-relevant event requiring immediate reassessment of the warming strategy.

Secondary endpoints included:

- central (rectal) and local temperature trajectories over time
- differences between local skin temperature and rectal core body temperature
- temperature changes from the onset of occlusion to the respective maximum or nadir (ΔT)
- frequency of threshold exceedance events for both skin and core temperature

Data were analyzed descriptively. Temperature data were extracted from the digital anesthesiology documentation system, compiled into a standardized database, and complemented by sternal infrared temperature measurements.

### 2.12. Ethics Approval and Patient Consent

Ethical review and approval were waived for this study, as the Ethics Committee of the Bavarian State Medical Association (BLÄK, file number 2025-1061) classified the project as a quality assurance measure conducted under routine clinical care and not requiring formal ethical approval under §15 of the Professional Code for Physicians in Bavaria.

Explanation of the anesthesiological procedure and safety management, including warming, temperature monitoring, and prevention of shivering and nausea, is part of the obligatory patient counseling prior to written informed consent. All temperature monitoring procedures (rectal probe, skin sensors, and occasionally infrared control of the upper body skin surface) are part of routine anesthesiological management in our institution since the introduction of forced-air warming, and the monitoring does not vary depending on the anatomical site of perfusion. As stated in the ethics approval, all procedures were part of routine clinical care rather than a study setting and were therefore covered by the standard informed consent obtained from all patients.

All data were collected and analyzed in anonymized form.

## 3. Results

Under continuous convective warming during IPC, no evidence of local overheating or heat accumulation was observed at any predefined skin measurement site during the occlusion phase. The predefined thermal alarm threshold of 39.5 °C for skin temperature was not reached or exceeded at any skin measurement site in any patient, and no safety-relevant events related to the warming therapy occurred. In contrast to physiological conditions with normal systemic circulation, core temperatures remained consistently lower than local skin temperatures despite externally applied warming.

A total of 31 patients undergoing IPC were included (17 male, 14 female). Procedures were performed in the abdominal (*n* = 16), thoracic (*n* = 12), and pelvic (*n* = 3) settings. Temperature measurements at all predefined sites were complete throughout the entire occlusion phase.

Maximum local temperatures remained within the normothermic range across all anatomical regions (see Table 2). The highest absolute single value was 37.7 °C (lumbar measurement site in the abdominal setting). Maximum local temperatures were lower during thoracic procedures. No progressive temperature increase or consistent pattern of local heat accumulation was observed.

**Figure 3:**
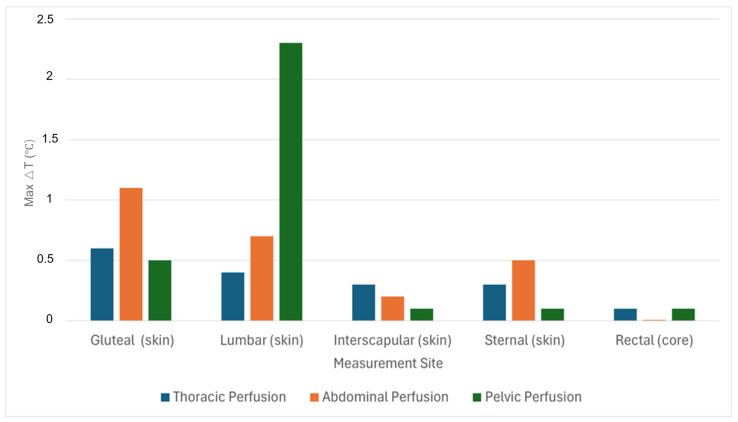
Maximum local temperature change (ΔT) at different measurement sites across perfusion settings.

Local skin temperature changes (ΔT) during the occlusion phase were generally small (mean ΔT: gluteal +0.12 °C [range −0.50 to +1.10 °C], lumbar +0.12 °C [range −1.00 to +2.30 °C], sternal +0.07 °C [range −0.10 to +0.50 °C], and interscapular +0.02 °C [range −0.40 to +0.30 °C]). The largest local skin temperature increase was +2.3 °C (lumbar) and occurred as an isolated observation. Changes at the rectal measurement site, used as the reference for core body temperature, were minimal (maximum +0.1 °C) (see Figure 3).

In most cases, local skin temperatures remained below rectal core body temperature. In 5 of 31 patients, local temperature exceeded core temperature at individual measurement sites; these differences were small, non-progressive, and attributable to comparatively low core body temperatures at the respective time points. Even in these cases, absolute local temperatures remained well below clinically relevant thresholds.

In addition, the standardized postoperative clinical examinations performed on the evening of surgery revealed no signs of thermal skin reactions at any assessed contact or support zone. No erythema, irritation, or patient-reported discomfort suggestive of thermal injury was observed.

## 4. Discussion

This prospective feasibility study evaluated the thermal safety of convective warming during transient vascular occlusion in Isolated Perfusion Chemotherapy. Under continuous multisite temperature monitoring, no evidence of local overheating or heat accumulation was observed.

This study should be interpreted within the framework of a pragmatic feasibility and safety assessment under real-world clinical conditions. Accordingly, temperature measurements were designed to capture the effective thermal exposure at patient–support contact zones rather than to isolate epidermal temperature as an independent physiological variable. This approach reflects the clinically relevant interface at which potential thermal injury would occur in the presence of reduced perfusion and directly addresses the primary safety concern of the study.

Concerns regarding warming during ischemia are primarily based on the absence of perfusion-mediated heat distribution and the potential for increased intracellular metabolic activity under hypoxic conditions. Blood flow normally acts as an efficient heat transport mechanism in biological tissue, whereas ischemia may impair this mechanism and theoretically allow local heat accumulation [25,28,29]. Local skin temperatures were therefore interpreted cautiously as an indirect indicator of potential heat accumulation in underlying tissues [30]. However, the present data do not support this concern under the clinical conditions investigated. Local temperature increases during occlusion were small, and absolute temperatures remained well below levels associated with thermal tissue injury. An isolated maximum increase at the lumbar site occurred from a comparatively low baseline temperature and remained well within the normothermic range, supporting the interpretation as a localized rewarming effect rather than clinically relevant heat accumulation. Core temperatures remained within the predefined safety range throughout the procedure, indicating no clinically relevant thermal exposure of internal organs during warming under hypoxic conditions. Several factors contributed to this observation. First, ischemic intervals were relatively short, ranging from five to fifteen minutes. Second, patients entered the occlusion phase with reduced peripheral temperatures due to prior heat loss, allowing moderate warming without reaching critical temperature levels.

Despite the theoretical concern that impaired perfusion might promote local heat accumulation, no clinically relevant temperature elevations were observed at any predefined skin measurement site or in core temperature measurements. At the same time, the study illustrates that warming alone cannot fully compensate for heat loss caused by extracorporeal circulation, hemofiltration, and adjunctive cooling strategies. This is consistent with current recommendations on perioperative temperature management, which emphasize a bundle of warming strategies adapted to individual patient needs and local conditions, including active surface warming (e.g., blankets and mattresses), warmed infusions, and structured checklist-based approaches [31]. Structured perioperative temperature management and monitoring therefore remain essential during IPC.

An additional aspect that should not be overlooked is the increasing shift toward interventional treatment strategies, including catheter-based chemotherapy and interventional radiology. Both approaches involve puncture of large-caliber vessels, making reliable hemostasis essential. In this context, hypothermia is of particular relevance, as it directly impairs coagulation. Experimental data demonstrate a temperature-dependent reduction in platelet function and prolongation of clot formation, indicating a clinically relevant impact even at moderate hypothermia [32].

These findings address a widely held precautionary concern regarding active warming during vascular isolation, where impaired perfusion is intuitively assumed to limit heat distribution and potentially increase the risk of local heat accumulation. Despite the lack of direct clinical evidence, this concern often results in a cautious or restrictive use of active warming in such settings and is reflected in safety warnings provided in manufacturer instructions for forced-air warming devices [33]. In contrast to this widely adopted precautionary approach, our data demonstrate that, under conditions of controlled application, close temperature monitoring, and short ischemic intervals, convective warming does not result in local overheating or thermal safety signals during transient ischemia.

While the present findings primarily address the safety of warming during transient ischemia, they need to be interpreted within the broader perioperative context of IPC. In this broader context, warming is not a “nice-to-have” but a necessary part of the treatment protocol and covers the entire perioperative period. Warming should be initiated upon entering the operating room and continued until the end of the recovery period.

Beyond the short ischemic interval itself, the overall perioperative context of IPC is characterized by repeated cooling exposure due to treatment protocols aimed at limiting the side effects of chemotherapeutic agents. The head cooling protocol alone includes approximately 45 min of pre- and post-cooling, in addition to the duration of IPC, the low ambient operating room temperature, and heat loss associated with hemofiltration of central blood volume.

As a result, patients are exposed to cumulative heat loss over a substantial period, placing them at considerable risk of progressive hypothermia. In this context, active warming should not be viewed as a short, procedure-specific intervention during the occlusion phase, but rather as a continuous perioperative strategy. The present data also argue against unnecessary interruption of warming in a setting where cumulative heat loss is substantial. This provides prospective clinical evidence in this specific setting and supports a reassessment of current temperature management strategies and may facilitate a shift toward standardized, actively managed normothermia in isolated perfusion chemotherapy. The present study should therefore be understood as a foundational feasibility step to establish a clinically acceptable safety baseline, representing a necessary prerequisite for subsequent investigations with larger sample sizes and extended outcome assessment.

## 5. Limitations

This study has several limitations. It represents a single-center feasibility assessment with a modest sample size. In addition, skin temperature was used as a surrogate marker for deeper tissue temperature and may not fully reflect intramuscular thermal conditions. Finally, ischemic intervals in the investigated procedures were relatively short, and the results cannot be extrapolated to prolonged ischemic conditions.

## 6. Conclusions

Within the clinical setting investigated, forced-air warming during balloon-induced transient ischemia did not result in local overheating or clinically relevant heat accumulation at either the skin or core temperature level. When combined with structured temperature monitoring, continued active warming appeared feasible, showed no thermal safety signal in this cohort, and may contribute to the prevention of perioperative hypothermia during Isolated Perfusion Chemotherapy (IPC).

## Figures and Tables

**Table 1:** Isolated anatomical regions and corresponding target malignancies.

Isolated Pelvic Perfusion	ovarian cancer, cervical cancer, rectal cancer; anal cancer, sarcomas
Isolated Abdominal Perfusion	Locally advanced primaries of gastric, colorectal, small bowel, pancreatic, renal, ovarian cancers, sarcomas and peritoneal carcinomatosis of those.
Isolated Thoracic Perfusion	lung cancer; breast cancer; pleural carcinomatosis; pleural mesothelioma, pulmonary metastases of various primary origins, sarcomas

**Table 2:** Highest measured temperatures (Tmax, °C) during the occlusion phase by procedural setting and measurement site.

Procedural Setting	Gluteal	Lumbar	Interscapular	Sternal	Rectal (Core)
Isolated Pelvic Perfusion	35.2	36.0	36.5	36.8	36.5
Isolated Abdominal Perfusion	36.2	37.0	37.7	38.2	36.8
Isolated Thoracic Perfusion	36.2	37.7	37.5	37.1	37.0

*Values represent the highest absolute temperatures recorded at each measurement site during the occlusion phase. No measurement approached the predefined thermal safety threshold for skin (39.5 °C), nor for the core temperature (37.5 °C)*.

## Data Availability

The data presented in this study are available on request from the corresponding author due to restrictions related to privacy and ethical considerations. The dataset is anonymized but not publicly available due to its complexity and structure and the potential risk of indirect re-identification.
